# Demirjian and Cameriere methods for age estimation in a Spanish sample of 1386 living subjects

**DOI:** 10.1038/s41598-022-06917-x

**Published:** 2022-02-18

**Authors:** Maria Melo, Fadi Ata-Ali, Javier Ata-Ali, José María Martinez Gonzalez, Teresa Cobo

**Affiliations:** 1 Department of Stomatology, Faculty of Medicine and Dentistry, Universitat de València, Valencia, Spain; 2 Department of Surgery and Medical-Surgical Specialities, Area of Orthodontics, Instituto Asturiano de Odontología, University Medical and Dental School, University of Oviedo (Spain), Oviedo, Spain; 3 Department of Dentistry, Faculty of Health Sciences, Universidad Europea de Valencia, Valencia, Spain; 4 Public Dental Health Service, Conselleria de Sanitat Universal i Salut Pública, Generalitat Valenciana, Valencia, Spain; 5 School of Dentistry, Complutense University of Madrid, Madrid, Spain; 6 Public Dental Health Service, Department of Hospital, Universitario y Politécnico La Fe, Avda. Fernando Abril Martorell, 106, 46026 Valencia, Spain

**Keywords:** Dentistry, Oral anatomy

## Abstract

Currently, human identification is a challenge. Migration due to war, economic crisis or other factors is frequent. The wisdom teeth are the last teeth to initiate and complete development therefore, are fundamental for determining the legal age of majority in different countries. The aim of the study is to determine the validity of two methods based on mineralisation of the third molar to predict the ages of majority of individuals in a Spanish population. Orthopantomographies of 636 men and 750 women (mean age, 16.5 years) were analysed. The Demirjian and Cameriere methods were used, and each tooth was assigned a value according to the degree of mineralisation and maturation. The level of significance used in the analyses was 5% (α = 0.05), with a power of 96.2%. The predictive ability of the Demirjian method to determine 18 years of age in the lower wisdom teeth 93%, respectively. The Cameriere method has a predictive capacity of 88%. There are no statistically significant differences between men and women. Stage H and a cut-off point of 0.08 were the guiding values for determining the age of majority of the study population. For other proposed age ranges (14 and 16 years), both methods were useful in determining the actual age of individuals, with the Demirjian method having a sensitivity of 97.5% with and Cameriere having a predictive capacity of 95%. Both methods can be used with high reliability to determine the age of individuals where reliable documentation is unavailable. Stage H with the Demirjian method and a cut-off point of 0.08 with the Cameriere method can determine the age of majority of the Spanish population. The combination of the two methods does not substantially increase predictive ability.

## Introduction

The estimation of an individual’s age is a necessary procedure in forensic medicine and for various legal matters such as identification of corpses, adoption procedures, illegal immigration, or determining legal liability^1–3^.

Several methods are available for determining the actual age of an individual, including those based on different stages of bone maturation, which have been proposed by different authors such as Fishman^4^, Björk^5^, or Bacetti^6^. However, several studies state that while these methods are useful in understanding growth potential, they are not useful in determining an individual’s age^7,8^. Methods based on the study of teething have proven to be more accurate in determining the actual age of an individual. They are based mainly on the different stages of calcification and are less influenced by external environmental factors^9–11^.

The Demirjian method consists of 8 stages (A–H). The first 4 (A–D) refer to the formation of the crown. The following stages (E–H) range from the beginning of root formation to apical closure^12^. This method tends to overestimate the actual age of individuals^13–15^, with a similar mean in men (0.65 years) and women (0.60 years)^16^. Intra- and inter-examiner concordance is good^1,17^, which implies that it can be used by the scientific community to determine the age of individuals. The Cameriere method also uses X-rays. A numerical value is obtained by dividing the sum of the widths of the intern margin of the two open apices by the length of the tooth^18^. Several studies have shown high precision with this method, which is between 72.4 and 75.6% for women^19^ and 94.5–97.6% for men^20,21^. The lowest precision value was found in a study by Deitos et al., with a value of 67.2% in men less than 18 years of age^22^.

There is no method for accurately knowing the actual biological age of an individual^23^. Timmins et al.^24^ state that to know the age of individuals who are supposedly between the ages of 7 and 16, the ideal scenario would be to perform the Demirjian and Cameriere methods and a study of cervical vertebrae maturation together. On the other hand, Galic et al.^25^ argue that the Cameriere method is the most accurate for determining age in individuals between 6 and 13 years old.

The wisdom teeth are the last teeth to initiate and complete development^26^ and are also the last morphological trait, useful to determine the age of an individual and determine the majority legal age in different countries. For example, the minimum age of criminal responsibility is 14 (Venezuela, Colombia), 16 (Argentina), or 18 (Spain, Brazil, Ecuador). In addition, the minimum age for sexual consent, marriage, or access to a job varies across different countries of the world^27^. According to different studies, the stage of the wisdom teeth can indicate, with sufficient precision, the probability that a person is at least 18 years old. Several studies have determined the relationship between the development of wisdom teeth and chronological age^28–30^. Marrero-Ramos et al.^28^ conducted a study on a sample of 180 patients with a mean age of 21.6 years and claimed that the Demirjian method, applied to the wisdom teeth, is necessary to determine whether an individual has reached 18 years or more, and presents high inter-examiner agreement. A study by Mwesigwa et al.^29^ on 1021 patients in Uganda, also confirmed that this is a valid method for determining age between 10 and 22 years. On the other hand, several authors determined that the Cameriere method applied to the wisdom teeth was valid for determining 18 years of age in different populations in the Dominican Republic^30^, Brazil^31^, Japan^32^, or Sardinia^33^. The degree of mineralisation of the wisdom teeth depends on and is specific to the studied population^34^.

There are very few studies that assess age determination in the Spanish population^35^ by means of the mineralisation stages of the wisdom teeth. The aim of the study is to determine the validity of Demirjian and Cameriere methods to predict the ages of individuals in Spanish population.

## Methods

A retrospective study of 1386 panoramic X-rays from Spanish patients between 10 and 26 years of age (636 men and 750 women) was carried out.

All patients underwent a panoramic X-ray study, as clinically indicated, at the dental clinic of the University of Valencia (Valencia, Spain) between 2010 and 2016. Informed consent was obtained from all subjects. In case of minors, it was obtained from a parent and/or legal guardian. An Orthopantomograph P100 (Instrumentarium Dental, Tuusula, Finland) was used for the X-rays. X-rays were randomly selected. An initial evaluation revealed that all the wisdom teeth were present in the X-rays^36^ and that they were of sufficient quality for the study. Radiographs of patients with chronic diseases, genetic alterations, signs of malnutrition, or of non-ASA-I status were excluded, along with those who did not meet the above inclusion criteria. The study was conducted in accordance with the declaration of Helsinki and was approved by the institutional review board of the University of Valencia (H1422858921172).

**Figure 1:**
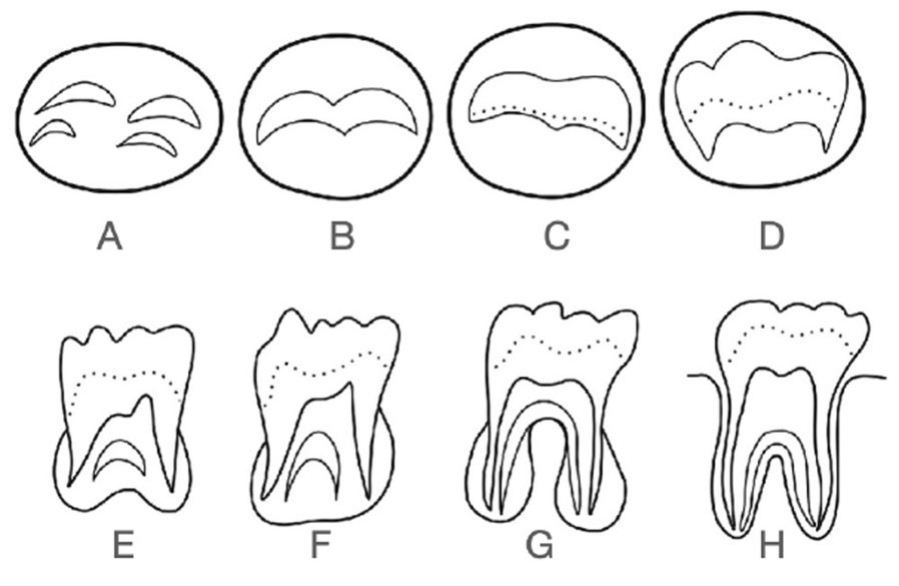
Representation of Demirjian method.

**Figure 2:**
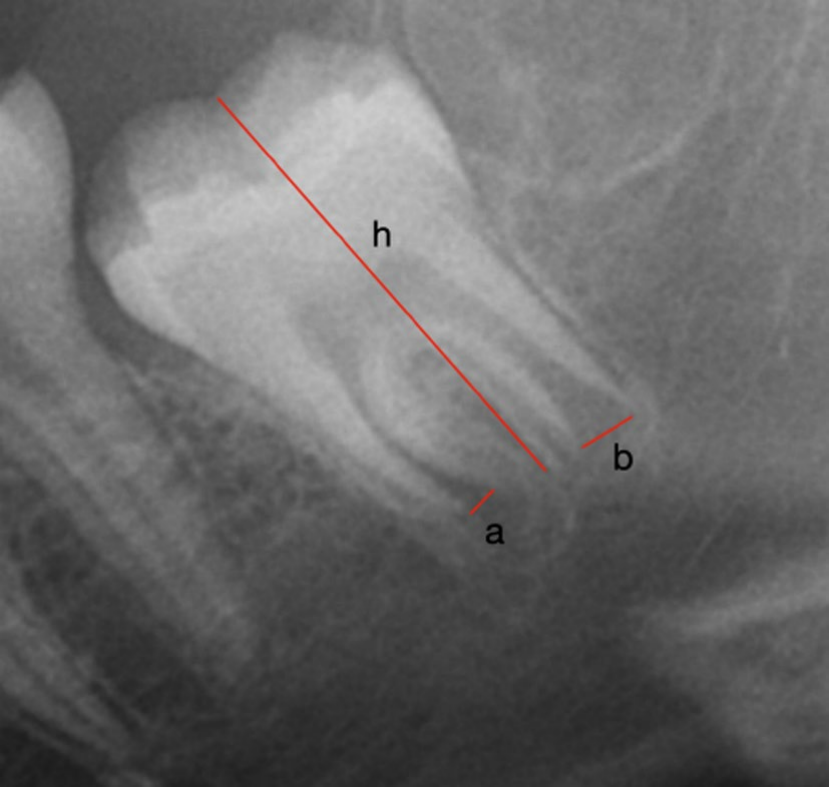
Representation of Cameriere method. The numerical value is obtained with the formula (a + b)/h.

The actual age of the individuals was calculated based on the date of birth on official documents and the time the X-ray was performed. The degree of development and mineralisation was collected with the Demirjian^12^ and Cameriere methods^37^. In the first method, each wisdom tooth was assigned a stage from A to H as follows: (A) First calcifications in the upper crypt portion. The calcification points are not fused. (B) Fusion of the calcification points forming one or more cusps. (C) Enamel formation on the occlusal surface, and beginning of dentin deposition. The pulp chamber has a curved shape in the occlusal margin. (D) Formation of the complete crown up to the cementoenamel junction. The upper part of the pulp chamber is curved in single rooted teeth and trapezoidal in multirooted teeth. Root formation begins. (E) Crown length is less than coronal height. The formation of a furcation in a semilunar form is observed, and the root length remains smaller than the coronal length. (F) The calcified furcal area is funnel-shaped and the length of the root is equal to or greater than that of the crown. (G) The walls of the root canal are parallel, and the apex is partially open. (H) The apex is completely closed. This method is represented in Fig. 1. The Cameriere method obtains a numerical value by dividing the sum of the widths of the internal margins of the open apices of that tooth by the length of the tooth, as it is explained in Fig. 2.

One observer (MM) performed observations, measurements, and data collection using both methods mentioned above. A second X-ray evaluation was performed 6 to 8 weeks after the first one to determine the intra-examiner reproducibility of each method. The observer was blinded to this second evaluation and was unaware of the previous record. After evaluating its reproducibility, the Cameriere method was used to calculate a single value based on the mean of the two measurements made, if a difference greater than 0.5 did not exist between them. If there were discrepancies between the two evaluations with the Demirjian method, the radiographs were re-evaluated along with a second observer (JAA) and the value or stage to be assigned in that case was determined.

The inferential analysis included: the t-test of independent measures (to compare the mean age of men and women with the same stage of maturation); the 1-way ANOVA F-test (to compare the mean age on 4-molar assessment in subjects of the same sex); and the Mann–Whitney test (to compare the distribution of maturation grades in men and women of the same age group). Simple linear regression analyses were established to explain and predict age, based on maturation status. Their predictive ability was assessed in terms of R^2^. The ages considered critical were 14, 16 and 18 years. To assess whether the degree of maturation of the third molar was a reliable parameter to establish age, a linear regression model was performed for each tooth. Kappa coefficient was used to assess intra and inter examiners’ reproducibility. The SPSS version 20.0 statistical package (Chicago, IL, USA) was used for statistical analysis. The level of significance used in the analyses was 5% (α = 0.05), with a power of 96.2%.

### Ethics approval

The study was conducted in accordance with the declaration of Helsinki and was approved by the institutional review board of the University of Valencia (H1422858921172).

## Results

The sample consists of 1386 subjects. The age and sex distribution of the total sample are described in Table 1.

**Table 1:** Age and sex distribution of the total sample.

	Sex					
	Total		Men		Women	
	N	%	N	%	N	%
Total	1386	100	636	100	750	100
10 years	103	7.4	47	7.4	56	7.5
11 years	102	7.4	48	7.5	54	7.2
12 years	146	10.5	64	10.1	82	10.9
13 years	110	7.9	48	7.5	62	8.3
14 years	88	6.3	46	7.2	42	5.6
15 years	90	6.5	54	8.5	36	4.8
16 years	108	7.8	40	6.3	68	9.1
17 years	108	7.8	48	7.5	60	8.0
18 years	105	7.6	50	7.9	55	7.3
19 years	125	9.0	61	9.6	64	8.5
20 yars	82	5.9	47	7.4	35	4.7
21 years	91	6.6	30	4.7	61	8.1
22 years	35	2.5	11	1.7	24	3.2
23 years	30	2.2	15	2.4	15	2.0
24 years	31	2.2	15	2.4	16	2.1
25 years	32	2.3	12	1.9	20	2.7

The sample comprised of individuals between 10 and 26 years of age, with a mean age of 16.5 years (Table 2).

**Table 2:** Age (years) according to sex.

	Sex		
	Total	Man	Woman
N	1386	636	750
Mean	16.51	16.42	16.58
Standard deviation	4.05	3.96	4.12
Minimum	10.00	10.00	10.00
Maximum	25.70	25.70	25.60
Median	16.50	16.30	16.50

The intra-examiner reproducibility is higher for Demirjian (0.875) than Cameriere method (0.81). The interexaminer reproducibility is 0.79.

The mean ages of mineralisation in various stages in the third molar, with their standard deviations and standard errors, are described in Table 3.

**Table 3:** Age according to Demirjian dental status by sex.

Demijian stages			Age															
			A		B		C		D		E		F		G		H	
			Mean	SD	Mean	SD	Mean	SD	Mean	SD	Mean	SD	Mean	SD	Mean	SD	Mean	SD
Sex	Men	DEM 18	10.51	0.41	11.56	0.52	12.97	0.74	14.18	0.79	16.01	0.78	17.86	0.87	19.04	1.25	22.17	1.94
		DEM 28	10.69	0.59	11.41	0.65	13.25	0.74	15.23	0.75	16.54	0.87	18.01	0.97	19.27	0.98	22.34	1.85
		DEM 38	11.01	0.70	12.28	0.83	13.80	0.87	15.67	0.83	17.49	0.97	18.43	0.97	19.93	0.76	22.39	1.87
		DEM 48	11.15	0.73	12.63	0.89	14.15	1.29	15.96	0.86	17.69	0.99	18.63	0.87	20.29	0.79	22.57	1.91
	Women	DEM 18	10.38	0.46	11.62	0.71	12.85	0.51	14.20	0.60	15.94	0.90	17.64	1.01	19.06	1.32	22.12	1.83
		DEM 28	10.87	0.3	11.83	0.89	13.01	0.52	14.73	0.82	16.33	0.81	18.12	1.09	19.34	1.22	22.18	1.79
		DEM 38	11.15	0.89	12.32	0.79	13.71	0.95	15.53	0.94	17.28	0.97	18.52	1.01	20.09	0.99	22.51	1.68
		DEM 48	11.25	0.93	12.52	0.80	14.71	1.40	15.68	0.97	17.91	0.99	18.54	1.01	20.15	1.00	22.51	1.69

*SD* standard deviation.

The differences of the mean age for a certain Demirjian maturation state according to sex were punctual. The predictive capacity of the Demirjian method for both men and women was set at 97.5% for all age ranges.

With respect to tooth 28 and stage B, the mean age of the men (11.4 years) was significantly lower than that of the women (11.8 years) (p < 0.001). For stage C, the mean age of the men (13.2 years) was significantly higher than that of the women (13.0 years) (p = 0.01). For stage D, the mean age of the men (15.2 years) was significantly higher than that of the women (14.7 years) (p < 0.001). For tooth 38 and stage C, the mean age of men and women was lower, 13.8 and 13.71 respectively (p = 0.011). For tooth 48 and stage C, the mean age of the men (14.2 years) was significantly lower than that of the women (14.7 years) (p = 0.011). For stage D, the mean age of the men (16 years) was significantly higher than that of the women (15.7 years) (p = 0.042).

In both sexes, the four teeth in the same maturation states do not correspond to individuals of the same age (p < 0.001). The only exception is stage H (p > 0.05).

The assessment of the degree of maturation in men and women at a certain age is shown in Table 4, indicating that the rate of maturation is more advanced in women.

**Table 4:** Grade of maturation in men and women, according to Demirjian method.

	Tooth 18	Tooth 28	Tooth 38	Tooth 48
10 years	0.919	0.009**	0.867	0.581
11 years	0.493	0.124	0.662	0.882
12 years	0.043*	0.003**	0.004**	0.036*
13 years	0.563	0.845	0.046*	0.744
14 years	< 0.001***	< 0.001***	< 0.001***	< 0.001***
15 years	0.955	0.540	0.861	0.536
16 years	0.196	0.003**	0.945	0.650
17 years	0.083	0.341	0.059	0.325
18 years	0.015*	0.655	< 0.001***	0.010*
19 years	0.008**	< 0.001***	< 0.001***	< 0.001***
20 years	< 0.001***	< 0.001***	0.122	0.055
21 years	0.603	0.603	0.603	0.010*
22 years	1.000	1.000	1.000	1.000
23 years	1.000	1.000	1.000	1.000
24 years	1.000	1.000	1.000	1.000
25 years	1.000	1.000	1.000	1.000

*p < 0.05; **p < 0.01; ***p < 0.001.

In men, a subject is more than 14 years of age, if presenting with at least stage E. Similarly, subjects may be more than 16 years of age with a probability of 97.5% if presenting with at least stage F, and more than 18 if presenting with stage H for tooth 18 (all of them with a probability of 97.5%). The same data are obtained for tooth 28, with a 94.2% probability. Stage G determines the critical age of 18 years in tooth 38, with a certainty of 93.6%. The critical age of 14 years is determined by stage D in tooth 48, with a 92.1% probability. The other stages are kept the same.

Women are more than 14 years of age if presenting with at least stage E. They are more than 16 years of age if presenting with at least stage G and more than 18 years of age if they present a stage H for tooth 18 (all of them with a probability of 97.5%). In teeth 28, 38 and 48, the stages of maturation that indicate 16 and 18 years are maintained, with stage E being the one that marks 14 years.

To verify the predictive validity of the Demirjian method for the prediction of age ≥ 18 years, contingency tables were made for all wisdom teeth in men and women (Table 5). The prediction rule establishes that, for 18 and 28, Demirjian stage ≥ G indicates that the subject is over 18 years old. For 38 and 48, the critical stage is F.

**Table 5:** Predictive validity (%) of the Demijiran stages for predicting age over 18 years in men–women.

	Tooth 18 (stage G)	Tooth 28 (stage G)	Tooth 38 (stage F)	Tooth 48 (stage F)	All
Sensitivity	85.7–85.3	83.1–76.2	90.0–93.7	88.7–86.4	99.1–93.7
Specificity	94.8–96.1	96.8–97.8	93.3–94.8	95.3–96.1	90.1–94.8
PPV	90.4–93.1	93.7–95.6	88.5–91.8	91.5–93.2	85.1–91.8
PNV	92.1–91.4	91.0–87.0	94.3–96.1	93.7–92.0	99.5–96.1
Correct totals	91.5–92.0	91.8–89.6	92.1–94.4	92.9–92.4	93.4–94.4

According to the Cameriere method, the mean age of men (21.6 years) was significantly lower than that of women (22.0 years) for tooth 38 in the range of 0–0.04. In the range of 0.08–0.3, this interpretation is reversed. The mean age of men (18.1 years) was significantly higher than that of women (17.8 years). For tooth 48, no relevant difference was detected.

According to the same method, the rate of maturation was more advanced in women. For 14 years and in tooth 48, 4.3% men had a Cameriere value of 0.9 or less, but the percentage rose to 19.1% for women of that age. Women presented more maturation at the age of 18. Only in certain age range is it observed that the maturity range is significantly different between men and women.

To verify the predictive validity of the Cameriere cut-off of 0.08 for the prediction of age ≥ 18 years presented by some authors, some contingency tables were made for teeth 38 and 48 in men and women. The total value obtained is more than 92% in men and 95% in women (Table 6).

**Table 6:** Predictive validity (%) of the Cameriere criterion (< 0.08) for predicting age over 18 years in men–women.

	Tooth 38	Tooth 48
Sensitivity	79.7–90.0	82.6–90.3
Specificity	100–100	99.5–98.9
PPV	100–100	99.0–98.1
PNV	89.0–93.5	90.3–94.2
Correct totals	92.3–95.7	93.1–95.6

The combination of both methods raises predictability to above 94% in all cases, so the gain is not statistically significant.

## Discussion

Various methods have been proposed for estimating the actual age of an individual based on the maturation of the third molar^10,12,38^. The importance of knowing the stage of maturation in the wisdom teeth lies in the fact that they are the only teeth whose roots continue to develop after 16 years of age^39^. The Demirjian method is proposed as a simple, reproducible, and highly concordant method within and between examiners^40^. On the other hand, the Cameriere method is highly precise^41^, and so it was considered interesting to carry out a study to estimate the real age of a Spanish population with these methods, with special emphasis on 18 years of age, the legal age in our country.

Numerous studies have evaluated differences in calcification associated with sex and race with the Demirjian^9,36,42,43^ and Cameriere methods^44,45^. In the paper presented by Qing et al.^36^, no statistically significant differences were observed between men and women, except for tooth 48 in stage E that occurred earlier in women. Sisman et al.^9^ analysed a population of 900 Turkish individuals, where a statistically significant difference between sexes was present in stages D and G, with maturation occurring earlier in men than in women. In contrast, in our study, the mean age of men was higher than that of women in all stages except for tooth 48 and stage C, where the mean age of men (14.2 years) was significantly lower than that of women (14.7 years) (p = 0.011). Thus, women have the fastest degree of maturation, as observed in other published studies. Rai et al.^46^, in their work on individuals in northern India, found statistically significant differences in stages D and G, stating that third molar formation occurred earlier in women. Another published study states that stages F to H in the maxilla and stages A and E in the jaw occur in the same manner^47^. Hofmann et al.^34^ observed that stage C mineralisation occurred earlier in women than in men (p = 0.024), although the age difference was very small. Other studies found no statistically significant differences between sexes with the Demirjian method regarding third molar maturation^48,49^.

According to the results derived from our study, women have earlier third molar maturation than men, with the Cameriere method. This result agrees with other publications^50^. However, other authors found earlier mineralisation in men rather than in women^44,45^. However, in some cases, significant differences only occurred in the mean ages with a value ≥ 0.08, except for those ranging from 0.7 to 0.9^22^. Finally, it should be noted that other authors found no differences between sexes regarding the development of the third molar^51^.

Stage H has been considered a key factor in numerous studies for determining 18 years of age. These results coincide with those obtained in our study, although in the case of tooth 38, it is stage G that establishes 18 years of age. The presence of stage H in the third molars indicates an older individual in countries such as Spain or Brazil according to different authors^52^. However, Roberts et al.^53^ state that these results are not entirely reliable since the mean age obtained in them is too broad. For stage H, the mean age was 17.9 years in a study by Soares et al.^54^.

For its part, the cut-off point of 0.08 could be decisive in determining older and younger individuals based on the results obtained from our study, by using the Cameriere method. This data coincides with that published by other authors such as Ribier et al.^44^, who carried out their work in a French population. Galic et al.^18^ conducted their study in a Croatian population, concluding that in those with values below 0.08, 94.5% of women and 96.5% of men were 18 years or older; therefore, this index can be used with high precision in the studied population. Khare et al.^45^ also consider this to be a valid method for differentiating between minors and adults in the Chinese population, with a cut-off point of 0.08. The same result was obtained by Tafrount et al.^55^ in a population from south-eastern France and by Palmela-Pereira et al.^56^ and Albernaz et al.^57^ in the Portuguese population.

## Conclusions

The predictive capacity of 97.5% of the Demirjian method and of up to 95% with the Cameriere method helps determine the actual age of individuals, considering that we have managed to establish values for the studied ages. Stage H and cut-off point of 0.08 are useful in estimating age above 18 for men and women in Spanish population.

## Data Availability

The datasets used and/or analyzed during the current study are available from the corresponding author on reasonable request.
